# The comparison of bone mineral density of femoral head between non-hip fracture side and hip fracture side

**DOI:** 10.1038/s41598-020-70144-5

**Published:** 2020-08-03

**Authors:** Xianlong Li, Yueyang Xu, Weilong Lin, Yongqian Fan

**Affiliations:** Department of Orthopedics, Huadong Hospital Affiliated To Fudan University, No. 221 Yan’an West Road, Jing’an District, Shanghai, 200040 China

**Keywords:** Bone, Osteoporosis

## Abstract

We aimed to analyze the associations of bone mineral density (BMD) of femoral heads, age and gender, and compare the differences in BMD between fracture side and non-fracture side by “3D Spine Exam Analysis” module in QCT Pro software. In this study, we identified patients who had undergone quantitative computed tomography (QCT) examinations between March 2016 and July 2018 and measured their trabecular volumetric BMD (vBMD) of femoral heads. This retrospective study enrolled 367 subjects. A total of 149 participants with images were randomly selected to verify the repeatability of this method. The relationship among the vBMD, age and gender was analyzed (n = 367), and the difference of vBMD between non-fracture side and fracture side were studied in subjects (n = 75) with low-energy hip fracture on one side and compared the image quality of bilateral hip joints. The intraclass correlation coefficients (ICCs) between the results measured by 2 operators and the results measured by the same operator showed excellent agreement (ICCs > 0.9). Multivariate regression equation of vBMD of femoral head, age and gender showed statistical significance (*P* < 0.05). vBMD showed negative correlation with age (P < 0.05), and showed no statistically significant relation with gender (P > 0.05). vBMD of non-fracture side was higher than that of fracture side, but the difference was statistically significant only at the middle layer (*P*_*middle*_ < 0.05). In conclusions, the vBMD of femoral head as measured by "3D Spine Exam Analysis" module in QCT Pro software showed good repeatability. The trabecular vBMD of femoral head was negatively correlated with age, and not related with gender. The vBMD of femoral head was higher on non-fracture side than that on the fracture side.

## Introduction

With the aggravation of social aging and prolongation of life expectancy in the population, age-related diseases are getting wider attention. Among these diseases, osteoporosis and osteoporotic fractures seriously affects the health and quality of life of the elderly, bringing heavy burden to society and their family^1,2^. According to a meta-analysis and systematic review, the prevalence of osteoporosis in China has been increasing from the past 12 years, affecting more than one-third of people of 50 years and older, and the prevalence of osteoporosis is increased with age and is higher in females when compared to males^3^. However, controversy still existed regarding the prevalence of osteoporosis due to gender differences^4–6^. Osteoporotic fracture, especially the hip fracture, is one of the most serious complications of osteoporosis in the elderly population. In most of the cases, symptoms do not appear until the occurrence of a broken bone, and even minor stress may induce fractures if the bone mineral density (BMD) is decreased. Therefore, the prevention and reduction of osteoporotic hip fractures are clearly among the major current and future challenges^1,7^. In a prospective European femoral fracture study, the authors found that BMD of the femoral head was the most powerful hip fracture discriminator^8^. Currently, there are relatively few in vivo quantitative computed tomographic (QCT) studies investigating the femoral head site, especially almost empty research on which side hip fracture will occur and whether it is related to the volumetric BMD (vBMD) of femoral head^9,10^. A study intended to investigate whether the regional difference in the density of 3D finite element model of the femur can be used to predict hip fracture site in elderly females, Lin Z.L. et al. found that the intertrochanteric region or femoral neck region with lower BMD is more prone to hip fracture of the type on the corresponding site^11^. Therefore, although the femoral head is not the site of osteoporotic fracture, it is of great value in predicting the risk of hip fracture.

Osteoporosis is diagnosed based on the results of areal BMD (aBMD) as measured by dual-energy X-ray absorptiometry (DXA), but the information of aBMD on the pathophysiology of hip fracture is limited. In recent years, QCT provided measures regarding the trabecular and cortical vBMD. QCT has become an increasingly important clinical research tool that is replaces DXA in future for diagnosing and treating osteoporosis. However, due to QCT software shortage, there are some limitations on hip measurement. The method for measuring lumbar vBMD through QCT Pro software ("3D Spine Exam Analysis" module) is used to measure the femoral head.

In this study, we first evaluated the repeatability by measuring the trabecular vBMD of femoral head by "3D Spine Exam Analysis" module in QCT Pro software, followed by examining the associations of age and gender with vBMD of femoral head, and the differences between vBMD of the non-hip fracture side and hip fracture side in patients with low-energy hip fractures. The present study intended to investigate whether the vBMD of femoral head can be used to predict hip fracture in elderly population.

## Materials and methods

### Study subjects

A search of our retrospective patient database was performed to identify patients with QCT examinations in our hospital between March 2016 and July 2018. The exclusion criteria are as follows: subjects with chronic diseases that affect bone metabolism, such as parathyroid disease, kidney disease, adrenal disease, and tumors; and subjects who have used drugs that affect bone metabolism, such as glucocorticoids, bisphosphonates, and parathyroid hormone analogues. Also subjects with a history of hip fracture were excluded. Consequently, a total of 367 subjects, aged between 35 and 99, were recruited. This study included 262 females (35–99 years old), 105 males (39–97 years old), and 75 individuals (61–99 years old) with only one sided low-energy hip fractures whose clear image can be obtained on both sides. The images of 149 subjects (53–97 years old) were randomly selected from 367 subjects. To prove the repeatability of this method, two experienced operators measured the right femoral heads at different time points under separate conditions and one of the operators measured the same femoral heads again after 1 week.

This study has been approved by the Ethics Committee of Huadong Hospital Affiliated to Fudan University. Informed consent was obtained from all study participants.

### QCT scan acquisition

QCT images were acquired using Siemens Somatom Definition 64-slice CT scanner (Siemens AG, Erlangen, Germany) with a calibration Mindways QCT phantom (Mindways Software Inc., Austin, TX, USA). Scan parameters for CT scanning were as follows: 120 kV, 150 mAs, 1 mm slice thickness, 500 mm field of view(SFOV), and 512 × 512 matrix in spiral reconstruction and standard reconstruction. The Mindways calibration phantom was placed beneath the hip and scanned simultaneously. Both the hips were scanned by placing the patient in supine position from the top of the acetabulum to 3 cm below the lesser trochanter.

### Image processing

**Figure 1:**
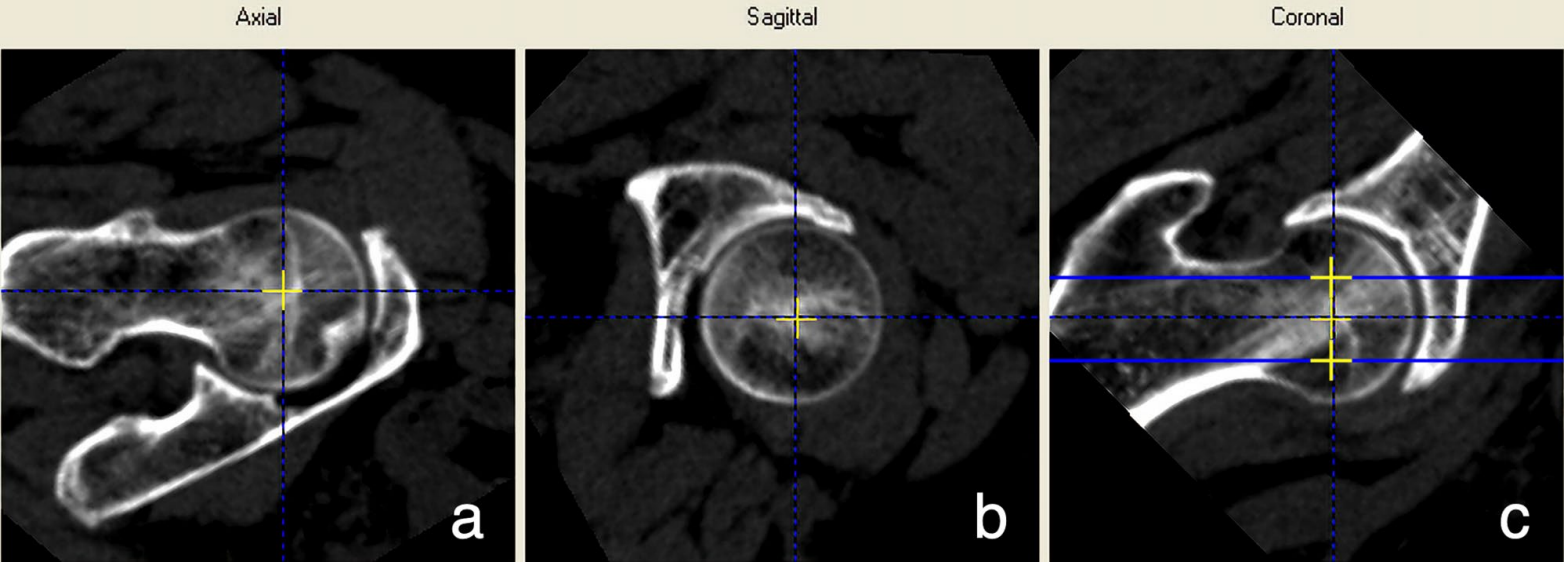
The image is manually adjusted in the QCT Pro software "3D Spine Exam Analysis" Software analytics module, (**a**) The horizontal line is parallel to the long axis of the femoral neck. (**b**) The yellow cross should be present in the center of the femoral head, (**c**) The horizontal lines are parallel to the long axis of the femoral neck. The upper, middle and lower layers are determined on the 3 planes that are parallel to the long axis of the femoral neck, and they are located close to the upper part of the femoral neck, the center of the femoral neck, and the lower part of the femoral neck.

**Figure 2:**
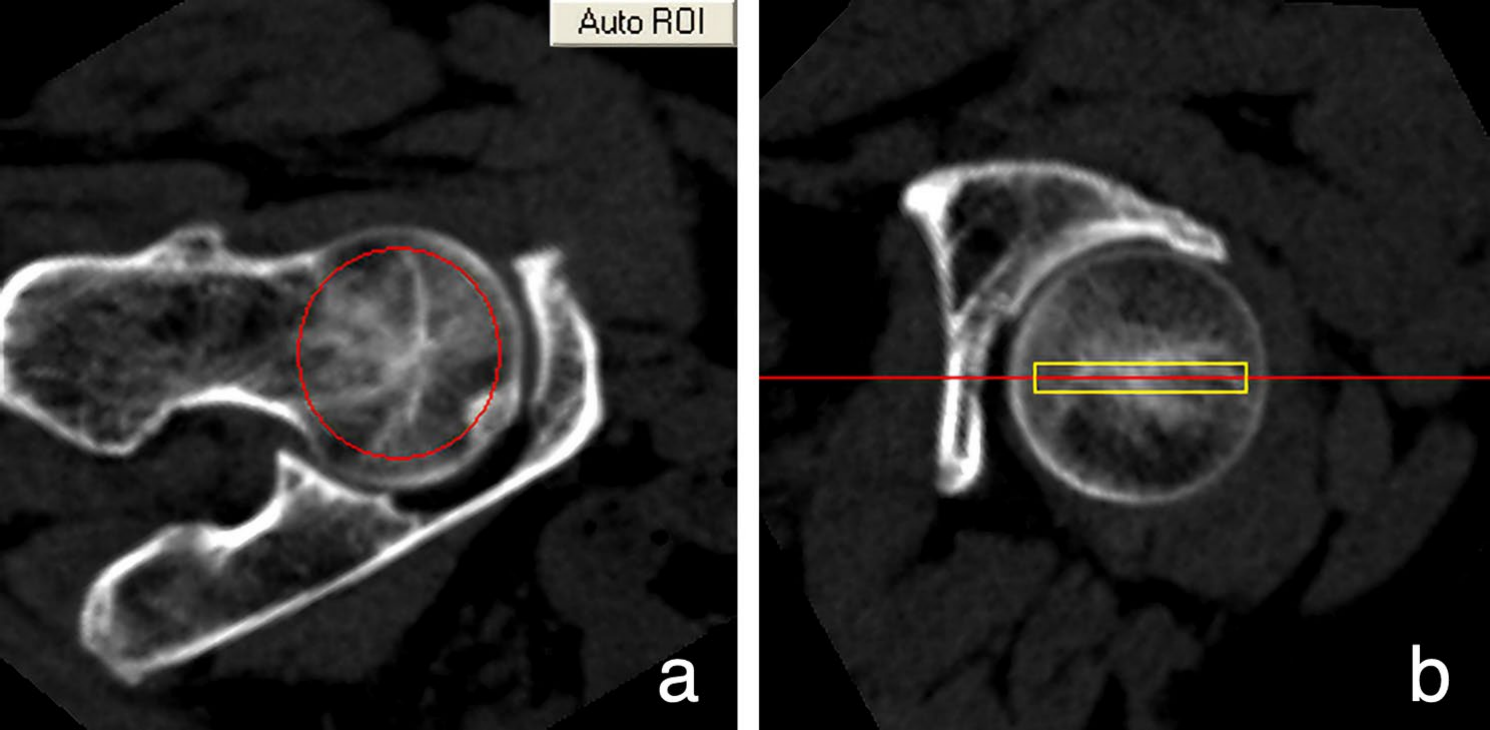
The ROI position is manually adjusted in the QCT Pro software "3D Spine Exam Analysis" Software analytics module. The ROIs containing the largest areas of the trabecular bone, without including the cortical bone.

The QCT data was then transferred to the QCT workstation and analyzed using the “3D Spine Exam Analysis” module of QCT Pro Version 4.2.3. The software automatically generates the reconstructed images of the proximal femur. Manual adjustment of the image was done by placing the axis of the femoral neck in the axial and coronal planes parallel to the horizontal line, in which the yellow cross should be present in the center of the femoral head on sagittal plane (Fig. 1). After that the 3 “Rotations” were determined on the coronal plane parallelly to the long axis of the femoral neck. To reduce the error caused by the “Rotations” settings, the femoral head was divided into 3 planes parallel to the long axis of the femoral neck: upper layer (close to the upper part of the femoral neck), middle layer ( through the center of the femoral neck) and lower layer ( close to the lower part of the femoral neck), (Fig. 1c). The regions of interest (ROI) were defined as oval-shaped areas of 5 mm thickness containing the largest areas of the trabecular bone, without including the cortical bone (Fig. 2). The vBMD values (mg/cm^3^) of the 3 layers were recorded and then were analyzed by 2 trained operators, respectively.

### Statistical analysis

Statistical analysis was performed using SPSS Statistics for Windows (version 24.0; IBM SPSS Inc., Chicago, IL). The normally distributed data were represented as $\stackrel{-}{x}$±s (standard deviation), otherwise by M (QR). (1) To study the repeatability of QCT measurement of the femoral head, the intraclass correlation coefficient (ICC) was performed to evaluate the repeatability between the results measured by 2 operators and between the results measured by one operator; (2) multiple linear regression was used to assess the multiple comparisons of age, gender and vBMD of femoral head ($${\mathrm{X}}_{\mathrm{gender}}=\left\{\begin{array}{c}0, female\\ 1, male\end{array}\right.$$); (3) for 75 patients with only one sided low-energy hip fractures and whose clear images can be obtained on both sides, a paired-sample t test was performed to compare the differences in vBMD between non-fracture side and fracture side. The trabecular vBMD of femoral head on non-fracture side and fracture side and along with the differences of femoral head between two sides of the same patient were checked for outliers and normality using Shapiro–Wilk tests. *P* < 0.05 was considered to be statistically significant.

### Ethical approval

All procedures performed in studies involving human participants were in accordance with the ethical standards of the institutional and/or national research committee and with the 1964 Helsinki declaration and its later amendments or comparable ethical standards.

### Informed consent

Informed consent was obtained from all individual participants included in the study.

## Results

The results of measuring vBMD of the trabecular bone of the femoral heads in 149 subjects by the 2 operators are presented in Table 1. Although the results in the same layer of the femoral head differed due to different operators, the difference was not statistically significant (*P* values were not less than 0.05). The ICCs of the results measured by 2 operators and the results measured by one operator showed excellent agreement in terms of upper, middle and lower layers (ICCs > 0.9) (Table 2). This suggested that different operators and the same operator using the "3D Spinal Examination Analysis" module in the QCT Pro software to measure the femoral head showed good repeatability.

**Table 1:** Results of 2 operators measuring the trabecular vBMD of femoral head.

Layer	Operator	Arithmetic Mean (mg/cm^3^)	Median (mg/cm^3^)	Inter-quartile range (mg/cm^3^)	Shapiro–Wilk test	Wilcoxon signed Rank test		Paired-sample T Test	
						Z	*P*	T	*P*
Upper	1	142.70 ± 36.34	139.73	(117.69, 164.84)	0.009	− 1.446	0.148	–	–
	2	140.92 ± 36.21	138.69	(114.70, 165.12)					
Middle	1	156.07 ± 42.61	157.78	(126.97, 177.22)	0.755	–	–	1.978	0.050
	2	154.83 ± 42.25	155.76	(124.25, 175.67)					
Lower	1	144.41 ± 44.45	146.60	(112.35, 171.69)	0.000	− 1.658	0.097	–	–
	2	142.43 ± 43.80	141.44	(111.20, 167.70)

**Table 2:** Intraclass correlation coefficient (ICC) between 2 operators and between results measured by the same operator.

	Layer	ICC	95% CI	*P*
Results measured by two operators	Upper	0.941	0.919–0.957	0.000
	Middle	0.983	0.977–0.988	0.000
	Lower	0.941	0.920–0.957	0.000
Results measured by the same operator	Upper	0.985	0.979–0.989	0.000
	Middle	0.996	0.994–0.997	0.000
	Lower	0.985	0.979–0.989	0.000

*CI* confidence interval.

The main results of multiple linear regression analysis of age, gender and vBMD of femoral head are presented in Table 3. The multiple linear regression equations in the 3 layers based on F-test showed a confidence level of 0.05 (*P* < 0.05). So, the regression equation was meaningful. The adjusted R square on the 3 layers of femoral head were ${R}_{Upper}^{2}=0.338, {R}_{Middle}^{2}=0.347,{R}_{Lower}^{2}=0.267$. It shown that the regression equation model has a good goodness of fit. The gender regression coefficient was greater than 0, in other words, the trabecular vBMD of males was higher than females. The results of “coefficients” showed a significant relationship between age and trabecular vBMD of the femoral head (*P* < 0.05), while there was no significant relationship between gender and trabecular vBMD of the femoral head (*P* > 0.05). There was no correlation between age and gender (Collinearity Statistics-VIF < 10). The results of this part indicate that the trabecular vBMD of the femoral head is related to age and has little relationship with gender (even if the trabecular vBMD of males was higher than females).

**Table 3:** Multiple linear regression results of vBMD of femoral head, age and gender.

Layer	ANOVA		Durbin–Watson	R	Adjusted R square	Coefficients-gender			Coefficients-age		
	*F*	*P*				Standardized coefficients Beta	*P*	Collinearity statistics-VIF	Standardized coefficients Beta	*P*	Collinearity statistics -VIF
Upper	94.328	0.000	1.778	0.584	0.338	0.043	0.313	1.006	− 0.579	0.000	1.006
Middle	98.347	0.000	1.697	0.592	0.347	0.072	0.089	1.006	− 0.582	0.000	1.006
Lower	67.585	0.000	1.707	0.520	0.267	0.049	0.280	1.006	− 0.514	0.000	1.006

The results of Shapiro–Wilk test are presented in Table 4. The trabecular vBMD of the femoral head in patients with hip fracture showed a normal distribution. The results of paired-sample t test in Table 5 showed that the trabecular vBMD of the femoral head on the fracture side was less than that on the non-fracture side in all the 3 layers, but the difference was statistically significant only in the middle layer (*P*_*Middle*_ = 0.000 < 0.05).

**Table 4:** Shapiro–Wilk test results of vBMD of patients with low-energy hip fracture.

Layer	Fracture side	Non-fracture side	Difference value
Upper	0.514	0.800	0.183
Middle	0.341	0.387	0.919
Lower	0.218	0.601	0.674

**Table 5:** Paired-sample T test results of patients with low energy hip fractures.

Layer	Side	Arithmetic mean (mg/cm^3^)	Standard deviation (mg/cm^3^)	Paired-sample T Test	
				*t*	*P*
Upper	Fracture side	134.56	33.48	− 0.941	0.350
	Non-fracture side	137.02	33.67		
Middle	Fracture side	139.38	31.54	− 4.340	0.000
	Non-fracture side	148.78	32.78		
Lower	Fracture side	130.98	34.59	− 1.662	0.101
	Non-Fracture side	135.95	26.60		

## Discussion

Osteoporosis is a metabolic disease with decreased bone strength and is characterized by reduced bone mass, increased skeletal fragility and deteriorated bone tissue structure. Osteoporosis is the most frequent cause of bone fractures in the elderly individuals. The bearing capacity of the femur is associated with many factors, such as internal (mainly refers to BMD and bone structure) and external factors. DXA is an X-ray based technique that is used to measure the aBMD of the lumbar spine or hip joint. However, the application of DXA in clinical work has the following disadvantages: (1) when measuring aBMD by DXA, the subjects are placed in a specific position. Patients with hip fractures or lumbar spine fractures are often difficult to sit in that position; (2) DXA is a 2D measurement that provides aBMD measurement results. It does not accurately measure the vBMD in a particular area, and it remains difficult to identify those patients who have aBMD increased osteoporosis due to degenerative lesions; (3) due to overlap of the acetabulum with the femoral head, the current ROI of DXA of hip does not include the femoral head. QCT is a 3D non-projection technique that calculates specific parameters, such as volume and density, and CT imaging data to quantify vBMD of the spine, proximal femur, forearm, and tibia. QCT is a powerful tool for measuring the quantity and quality of the bone, and it has the advantages over other density measurement techniques: (1) It can clearly distinguish cortical bone and trabecular bone; (2) the density of trabecular bone in ROI is less affected by the degenerative changes; (3) it can provide information such as 3D anatomical structure and volumetric density. According to the Guidelines for Diagnosis and Treatment of Primary Osteoporosis in China: Update 2017, the use of QCT has been recommended to measure the vBMD of lumbar for predicting the risk of vertebral fractures in postmenopausal women and to analyze the effect of drug therapy. Similarly, for measuring the vBMD of hip, it is important to predict the risk of hip fractures and the effect of drug therapy on the hip. It has been suggested that the lumbar BMD is less accurate than hip BMD as the lumbar BMD might be higher than the actual BMD due to degenerative changes or vascular calcification, and the femur neck or total hip BMD acts as a fracture risk factor more directly than the lumbar spine BMD in hip fracture^2^. There is a growing evidence that the femoral head, although not a site of osteoporotic fracture, is a powerful discriminator of hip fracture risk, and the conclusion was confirmed by Valérie Danielle Bousson et al.^8^. At present, QCT is regarded as the best way to evaluate vBMD of the femoral head. However, due to software limitations, the ROI of the vBMD measurements of the femoral head remained mostly perpendicular to the long axis of the femoral neck^10^^,^ while most of the studies used on femoral head were in vitro. But these studies showed limited significance for clinical guidance. The “3D Spine Exam Analysis” module in the QCT Pro software has higher space operability. Therefore, the “3D Spine Exam Analysis” module was used to measure the trabecular vBMD in different layers parallel to femoral neck axis of the femoral head. Operation procedure is similar to the procedure for measuring lumbar vBMD.

In this study, 2 operators measured the trabecular bone vBMD of the femoral heads of randomly selected 149 subjects. The ICC values for upper, middle and lower layers were excellent. This proved that the “3D Spine Exam Analysis” module in QCT Pro software was highly repeatability in measuring the femoral head. Although the results are not stable due to manual placement of ROIs, there were no significant differences in the measurements among different operators after training. This provided a new method for future research on the femoral head.

Trabecular bone is about 8 times more metabolically active than cortical bone. Age is considered as one of the most important factors affecting the trabecular bone density^2,12^. Generally, women suffer from osteoporosis more often than men, but there is still a controversy regarding the impact of gender differences on BMD^13–15^. According to a study that evaluated the relationships between menopausal statuses, hormone replacement therapy, body mass index, percent body fat, and exercise with osteoporosis and BMD in Singaporean women, the results of accelerated bone loss was not evident in postmenopausal women and that the loss of bone mass in postmenopausal women could be accounted to age alone^6^. Fjola Johannesdottir demonstrated that the loss in trabecular BMD was similar among men and women, but women loose more cortical vBMD than men^5^. In our study, it was found that the trabecular vBMD of femoral head was negatively correlated with age (the standardized beta coefficients of age on the upper, middle, and lower layers were -0.579, -0.582, and -0.514, respectively), and the correlation was statistically significant (*P* < 0.05), The gender regression coefficient was greater than 0, in other words, the trabecular vBMD of males was higher than females, but this correlation was not statistically significant (*P* > 0.05). The reason for this might be due to that the baseline levels of BMD of females were lower than that of males. In this study, the changes of trabecular vBMD in the femoral head were negatively correlated with age, and similarly there was no significant correlation observed with gender. This concluded that the change in trabecular vBMD of femoral head was mainly influenced by age rather than gender.

There is contradictory information on whether the rate of BMD loss is an independent risk factor for osteoporotic fractures and whether this should be included in the fracture prediction systems^16^. However, most of the studies still supported that the change in BMD in the femoral neck or total hip joint is considered as an important risk factor for hip fractures in the elderly^17,18^. At present, due to various limitations, there are few studies regarding the comparison of BMD between the non-fracture side and fracture side in the same patients with low-energy hip fractures^9^. In recent years, QCT has allowed precise measurement of hip structure non-invasively in vivo. In our study, it was found that the trabecular vBMD of femoral head on the fracture side was less than that on the non-fracture side, but the difference was statistically significant only at the middle layer. In one study, Chen Yi et al. quantified differences in trabecular BMD of the femoral head between patients with proximal femoral fractures and healthy subjects in the control group by using QCT. In their study, it was found that for control group, results showed no marked difference of BMD between left and right femur head (*P* > 0.05). And BMD of femur head in the fracture group was obviously lower than that in the control group (*P* < 0.05)^10^. Therefore, the measurement of trabecular vBMD in the middle layer of femoral head in the elderly can be helpful for predicting on which side of the initial low-energy hip fracture occurred. It can help prevent the occurrence of hip fractures at the same time. DXA is now widely used to diagnose osteoporosis, but it usually measures only one side of the patient's hips in China^19^. According to this study, we believed that the measurement of only one-side hip by DXA for diagnosing osteoporosis has a major flaw.

There are some limitations to our study, which were as follows: (1) the sample size and the number of first-time low-energy hip fractures that are included for analysis are insufficient. Therefore, it is necessary to further enlarge the sample size and improve the representativeness of the sample to verify the conclusion of this study. (2) There were some errors because of the manual operation performed during the operation. Hence, it is necessary for the technicians to optimize the software in order to reduce the errors.

In conclusion, trabecular vBMD of femoral head as measured by "3D Spine Exam Analysis" module in QCT Pro software showed good reproducibility. The trabecular vBMD in the femoral head was negatively correlated with age, but showed no significant correlation with gender. The first-time low-energy hip fractures more likely occur at the hip with low trabecular vBMD. Measurement of middle layer of femoral head by QCT Pro software can be a powerful tool for predicting on which side the first-time low-energy hip fracture might occur.
